# RuBisCO activity assays: a simplified biochemical redox approach for in vitro quantification and an RNA sensor approach for in vivo monitoring

**DOI:** 10.1186/s12934-024-02357-6

**Published:** 2024-03-14

**Authors:** Muhammad Faisal, Aditya P. Sarnaik, Nandini Kannoju, Nima Hajinajaf, Muhammad Javaid Asad, Ryan W. Davis, Arul M. Varman

**Affiliations:** 1 Chemical Engineering, School for Engineering of Matter, Transport and Energy (SEMTE), Arizona State University, Tempe, AZ 85281 USA; 2 University Institute of Biochemistry and Biotechnology, PMAS-Arid Agriculture University Rawalpindi, Rawalpindi, 46000 Pakistan; 3 Sandia National Laboratories, Livermore, CA USA

**Keywords:** Biosensor, Cyanobacteria, RNA aptamer, Calvin cycle, Fluorometric assay, Carbon capture

## Abstract

**Background:**

Ribulose-1,5-bisphosphate carboxylase/oxygenase (RuBisCO) is the most abundant soluble protein in nature. Extensive studies have been conducted for improving its activity in photosynthesis through approaches like protein engineering. Concurrently, multiple biochemical and radiolabeling assays have been developed for determining its activity. Although these existing assays yield reliable results, they require addition of multiple external components, rendering them less convenient and expensive. Therefore, in this study, we have developed two relatively cheaper, convenient, and easily reproducible assays for quantitative and qualitative estimation of RuBisCO activity.

**Results:**

We simplified a contemporary NADH based spectrophotometric RuBisCO assay by using cyanobacterial cell lysate as the source for Calvin cycle enzymes. We analyzed the influence of inorganic carbon substrates, CO_2_ and NaHCO_3_, and varying protein concentrations on RuBisCO activity. Ribulose-1,5-bisphosphate (RuBP) consumption rates for the cultures grown under 5% CO_2_ were 5–7 times higher than the ones grown with 20 mM NaHCO_3_, at different protein concentrations. The difference could be due to the impaired activity of carbonic anhydrase in the cell lysate, which is required for the conversion of HCO_3_^−^ to CO_2_. The highest RuBisCO activity of 2.13 nmol of NAD^+^/ µg of Chl-a/ min was observed with 50 µg of protein and 5% CO_2_. Additionally, we developed a novel RNA-sensor based fluorescence assay that is based on the principle of tracking the kinetics of ATP hydrolysis to ADP during the conversion of 3-phosphoglycerate (3-PG) to 1,3-bisphosphoglycerate (1,3-BPG) in the Calvin cycle. Under in vitro conditions, the fluorometric assay exhibited  ~ 3.4-fold slower reaction rate (0.37 min^−1^) than the biochemical assay when using 5% CO_2_. We also confirmed the in vivo application of this assay, where increase in the fluorescence was observed with the recombinant strain of *Synechocystis* sp. PCC 6803 (SSL142) expressing the ADP-specific RNA sensor, compared to the WT. In addition, SSL142 exhibited three-fold higher fluorescence when supplemented with 20 mM NaHCO_3_ as compared to the cells that were grown without NaHCO_3_ supplementation.

**Conclusions:**

Overall, we have developed a simplified biochemical assay for monitoring RuBisCO activity and demonstrated that it can provide reliable results as compared to the prior literature. Furthermore, the biochemical assay using 5% CO_2_ (100% relative activity) provided faster RuBP consumption rate compared to the biochemical assay utilizing 20 mM NaHCO_3_ (30.70% relative activity) and the in vitro fluorometric assay using 5% CO_2_ (29.64% relative activity). Therefore, the absorbance-based biochemical assay using 5% CO_2_ or higher would be suitable for in vitro quantification of the RuBisCO activity. On the other hand, the RNA-sensor based in vivo fluorometric assay can be applied for qualitative analysis and be used for high-throughput screening of RuBisCO variants. As RuBisCO is an enzyme shared amongst all the photoautotrophs, the assays developed in this study can easily be extended for analyzing the RuBisCO activities even in microalgae and higher plants.

**Supplementary Information:**

The online version contains supplementary material available at 10.1186/s12934-024-02357-6.

## Background

Ribulose-1,5-bisphosphate carboxylase/oxygenase (EC 4.1.1.39, RuBisCO) is the most abundant soluble protein found amongst the three different domains of life- archaea, bacteria, and eukaryotes. Since its discovery in 1947, four different organizational isoforms of RuBisCO (I; II; III; and RuBisCO like protein, RLP or form IV) have been identified, where the hexadecameric form I RuBisCO exists in most of the plants, algae, proteobacteria, and cyanobacteria [1, 2]. RuBisCO catalyzes the biological CO_2_ fixation through carboxylation of ribulose-1,5-bisphosphate (RuBP) in photoautotrophs [2–5]. However, RuBisCO activity is slow and non-specific to some extent, as it wastefully reacts with oxygen at relatively higher partial pressure, leading to its oxygenase activity, causing release of previously fixed CO_2_, NH_3_, and energy [4, 6, 7]. Recognizing the indispensable role of RuBisCO in photosynthesis, significant attempts have been made towards increasing its efficiency using molecular engineering strategies in both prokaryotic and eukaryotic photoautotrophs. [8–14].

With an increasing interest in engineering RuBisCO [15], there is an urgent need to develop a RuBisCO activity assay that is not only cheaper and faster but also convenient and reliable [15]. Previously, Rasmussen et al. had developed a biochemical assay for estimating RuBisCO activity from the permeabilized *Synechocystis* sp. PCC 6803 (hereafter PCC 6803) and *Synechococcus* sp. PCC 7002 cells which also required the addition of external enzymes and substrates. They demonstrated that the enzymatic activity observed in the permeabilized cells was higher than those observed in the cell-free lysates [16]. Recently, Sales et al. compared four different RuBisCO activity assays (glyceraldehyde-3-phosphate dehydrogenase (GAPDH)- glycerolphosphate dehydrogenase (GlyPDH) assay, phosphoenolpyruvate carboxylase (PEPC)-malate dehydrogenase (MDH) assay, pyruvate kinase (PK)- lactate dehydrogenase (LDH) assay, and a radiometric ^14^C-based assay) by using RuBisCO isolated from wheat plant [5]. The first three spectrophotometric assays used NADH-specific enzymes to follow the 4-step metabolic pathways to oxidize NADH: 1) 3-phosphoglycerate (3-PGA) to glycerol-3-phosphate (GAPDH-GlyPDH assay), 2) 3-PGA to malate (PEPC-MDH assay), 3) 3-PGA to lactate (PK-LDH assay). In all these 3 assays, the individual pathway enzymes were externally added to the reaction mixture, to monitor the NADH oxidation kinetics which was not directly resulting from the Calvin cycle. On the other hand, the radiometric RuBisCO activity assay used NaH^14^CO_3_ to trace the acid-stable ^14^C labelled products obtained from the biomass, by using a scintillation counter. Amongst all, the radiometric assay exhibited relatively higher sensitivity, and PK-LDH assay had the overall strongest correlation with ^14^C-based radiometric assay [5]. In other works; Atsumi et al., and Liang et al. have performed a similar ^14^C-based assay, while determining RuBisCO activity from a crude lysate of PCC 6803 [2, 9]. However, all the biochemical assays mentioned above require the addition of multiple non-cellular or external chemicals, including enzymes, intermediate metabolites and/ or buffer components [5, 16]. All these factors together would impact the assay cost, reproducibility, and accessibility for the user. On the other hand, radiometric assays have other major constraints such as generation of radioactive waste and the need for special protocols and lab space for handling radiolabeled C substrate [5]. Understanding these limitations, we endeavored to develop an NADH-based quantitative biochemical assay using native cyanobacterial enzymes present in the cell lysate, thereby minimizing the need for external reaction components. We further optimized the crude cell lysate protein concentration required for the reaction mixture containing NaHCO_3_ or CO_2_ as the inorganic carbon substrates.

**Fig. 1:**
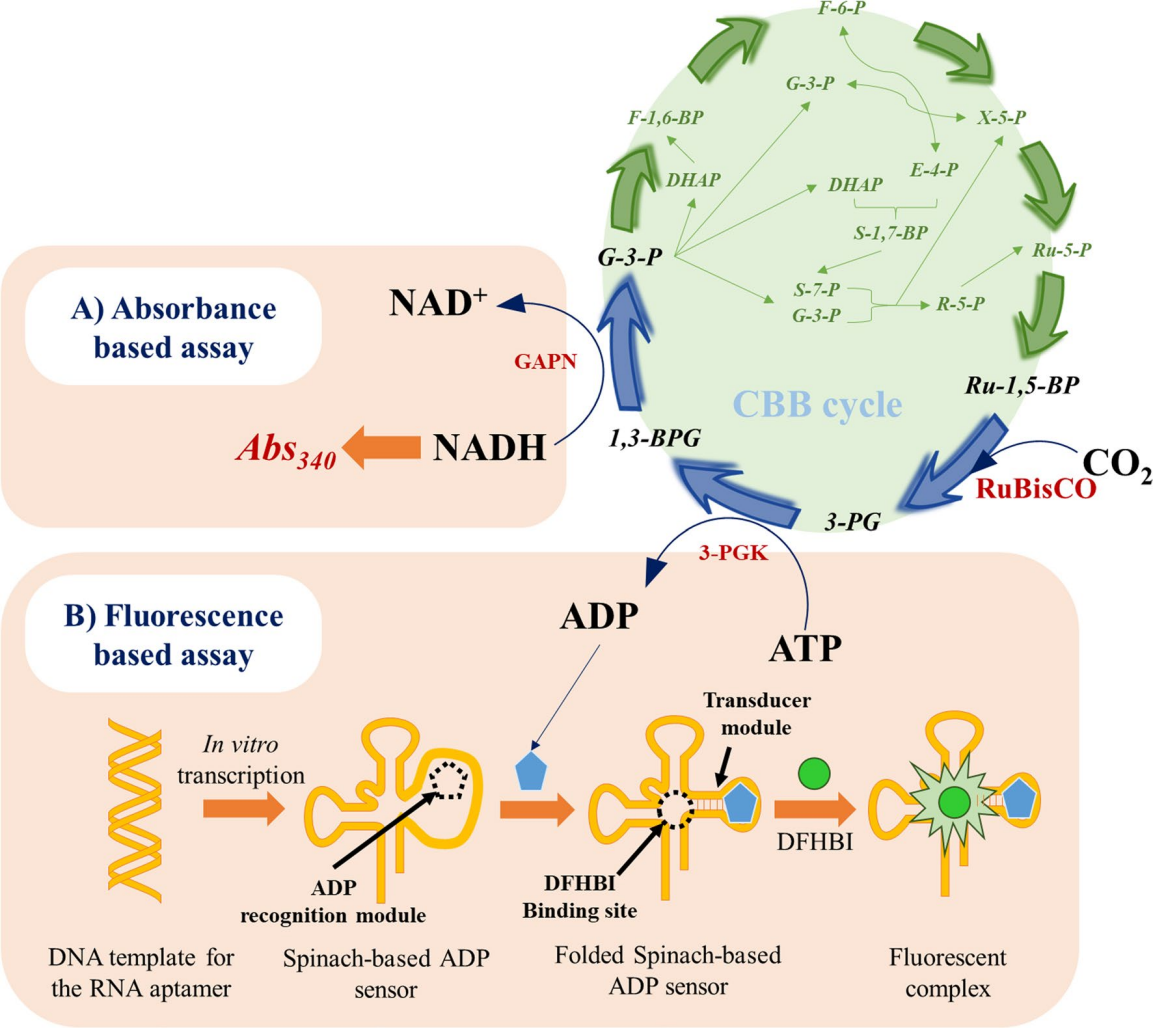
Schematic representation of the principles behind the RuBisCO activity assays. The kinetics of the reactions downstream of RuBisCO in the Calvin cycle were monitored to provide RuBisCO activity indirectly. Two distinct assays were developed, **A** an absorbance based assay in which the kinetics of NADH oxidation was monitored by measuring its absorbance at 340 nm [16], and **B** RNA sensor-based fluorescence assay using an RNA aptamer which is a Spinach-based ADP sensor. ADP resulting from ATP hydrolysis within the Calvin cycle binds to the recognition module, following which the RNA sensor folds allowing DFHBI binding resulting in a highly fluorescent Spinach-DFHBI complex [17]. 3-PG, 3-phosphoglycerate; 3-PGK, 3- phosphoglycerate kinase; 1,3-BPG, 1,3-bisphosphoglycerate; RuBP, ribulose-1,5-bisphosphate; G-3-P, glyceraldehyde-3-phosphate; GAPN, glyceraldehyde-3-phosphate dehydrogenase; CBB, Calvin-Benson-Bassham cycle

In addition, we have also developed a novel assay that leverages an RNA-aptamer based ADP-specific sensor for providing qualitative analysis of the RuBisCO activity. The presence of the ligand ADP in the reaction mixture induces folding in the RNA-aptamer, thereby creating a DFHBI (3,5-difluoro-4-hydroxybenzylidene imidazolenone) binding domain. DFHBI is a small molecule that exhibits properties similar to the chromophore of green fluorescent protein and gets activated to generate fluorescence after binding to the ADP-specific RNA aptamer [17]. In principle, the RNA-aptamer based ADP-specific sensor could track ADP produced from ATP hydrolysis during the conversion of 3-phosphoglycerate (3-PG) to 1,3-bisphosphoglycerate (1,3-BPG), catalyzed by 3-phosphoglycerate kinase (3-PGK). As 3-PG is the direct product of the RuBisCO catalyzed reaction, this assay involves a fewer number of reactions compared to the NADH-dependent assay (Fig. 1). The model cyanobacterium PCC 6803 was used as the source for RuBisCO to demonstrate the application of our technique(s), in vitro as well as in vivo. Moreover, detailed studies have been conducted to independently optimize both the biochemical and the fluorometric assays, for obtaining faster, precise, and reliable RuBisCO activity.

## Results and discussion

Multiple attempts have been made to improve the efficiency of RuBisCO, especially through protein engineering [12, 15, 18, 19]. However, the existing biochemical assays for estimating the efficiency of the engineered RuBisCO, demand the addition of external reaction components, especially enzymes, that ultimately increase the cost, upsurge the possibility of introducing manual errors, and affect robustness and reproducibility. Therefore, this work presents a relatively simple and cost-efficient RuBisCO activity assay utilizing a major proportion of cellular metabolites and enzymes, and requiring minimal addition of external reaction components.

### NADH-dependent RuBisCO activity assay

First, we developed a biochemical assay which was based on the principle of tracking the kinetics of NAD(P)H oxidation by glyceraldehyde-3-phosphate (G-3-P) dehydrogenase (GAPN), that will lead to the estimation of corresponding RuBisCO activity (Fig. 1). Prior to employing RuBisCO in carboxylation reactions, a post-translational modification of RuBisCO was required for it to be activated. Activation of RuBisCO involves the reaction of a CO_2_ molecule with a lysine residue within its active site to produce a carbamate, which is then quickly bound by an Mg^2+^ ion to produce an activated ternary configuration [1, 20]. Henceforth, a 20-min pre-incubation or carbamylation of the protein under 5% CO_2_ was performed for complete RuBisCO activation. The CO_2_ fixation reaction would then be carried by RuBisCO through the following reactions in series: (1) the first step is RuBisCO catalyzed RuBP carboxylation to 3-PG; (2) the second step is 3-PGK catalyzed phosphorylation of 3-PG to 1,3-BPG; and (3) the third step in which 1,3-BPG is converted to glyceralehyde-3-phosphate (G-3-P) by virtue of GAPN catalysis while oxidizing NADPH to NADP^+^. Amongst these three, RuBisCO catalysis is considered as a major bottleneck in photosynthesis [2, 18, 21]. A recent kinetic study by Sporre et al. has also shown that RuBisCO exhibits a higher positive flux control over the reactions catalyzed by 3-PGK and GAPN in PCC 6803 [22]. Therefore, as RuBisCO is the bottleneck enzyme in this pathway, we hypothesized that measuring the kinetics of NADPH oxidation at high substrate concentration for RuBisCO would provide an indirect estimation for RuBisCO activity [5, 16]. However, considering the instability of NADPH under the reaction conditions, NADH was used as an alternative cofactor, that has provided reliable activity in previous studies [16]. In addition, ATP was externally supplemented under all the reaction conditions. The reliability of using NADH to monitor RuBisCO activity was confirmed by performing in vitro enzyme assays with and without the external supplementation of RuBP and/or NADH (Additional file 1: Fig. S1). NADH oxidation was observed in the presence of RuBP supplementation. However, the control condition with NADH but without RuBP supplementation exhibited negligible increase in the absorbance, confirming minimal interference from background reactions. Although the contribution of other metabolic pathways towards NADH oxidation was low in this assay, it can increase when the assay gets extended to other strains. For example, extending the NADH-based assay for analyzing the RuBisCO activity in a recombinant cyanobacterial strain overexpressing heterologous enzymes performing NADH oxidation, might contribute to the background NAD^+^ pool. However, this limitation can be circumvented by adding RuBP to the reaction mixture, which would increase the rate of NADH oxidation due to photosynthetic C-fixation (Additional file 1: Fig. S1). Hence, our assay traced NADH oxidation kinetics that occurred due to the presence of GAPN in the crude cell lysate.

**Fig. 2:**
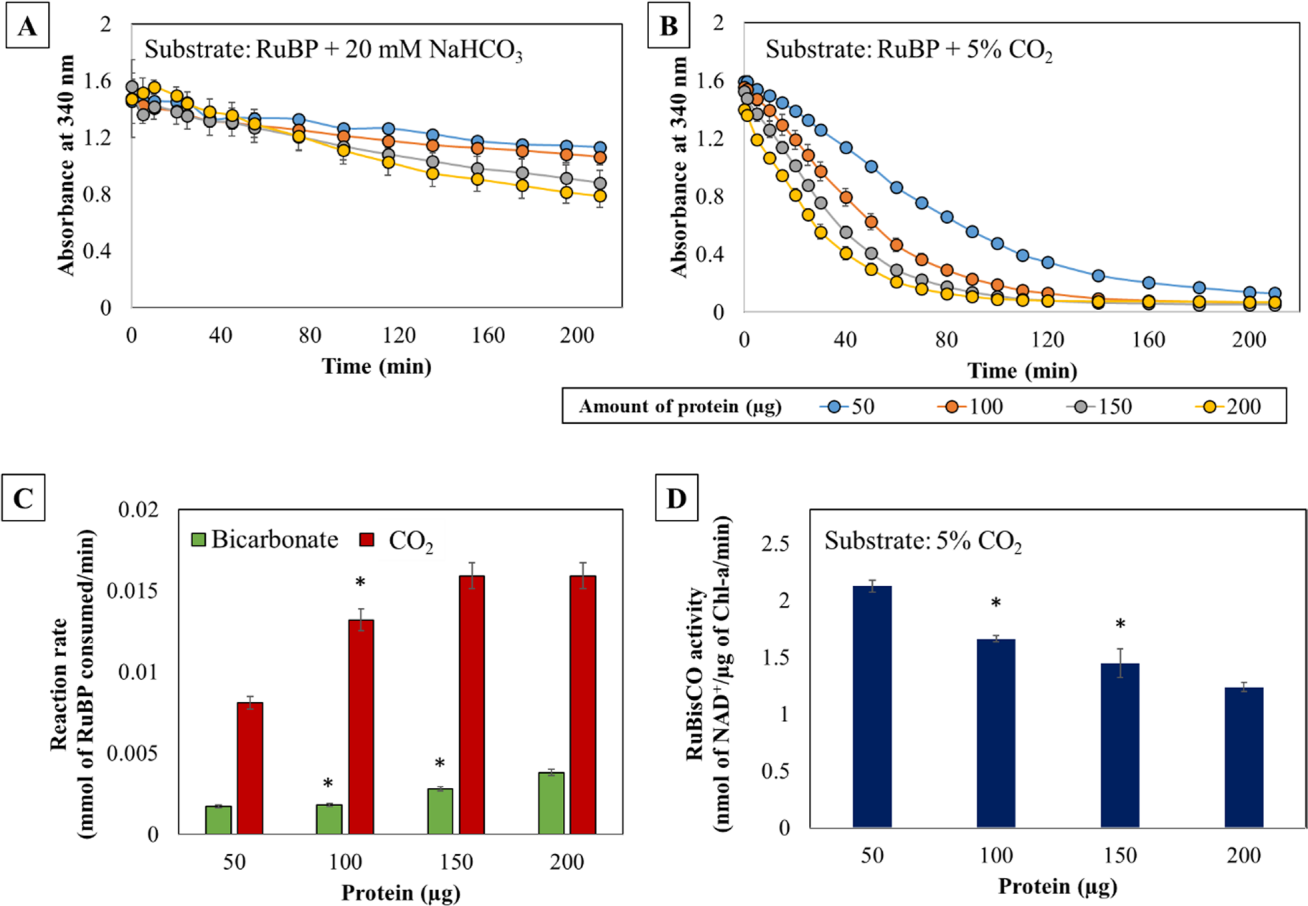
RuBisCO activity was estimated through an NADH-based assay using two inorganic carbon substrates, **A** NaHCO_3_, **B** CO_2_, in conjunction with RuBP, and varying amounts of crude protein (50, 100, 150, 200 µg). **C** Reaction rates were calculated as mmol of RuBP consumed per minute and compared between reactions supplemented with CO_2_ and bicarbonate as inorganic C substrates. The reaction rates supplemented with CO_2_ were significantly higher than those for bicarbonate. **D** Comparative analysis of RuBisCO activities with CO_2_ as a substrate, estimated in terms of photosynthetic NADH oxidation per µg of Chl-a per minute, as a function of variable amounts of cyanobacterial crude protein. The results indicated that the highest activity was observed with 50 µg of the total protein. All the assays were performed in biological triplicates and statistically significant differences (presented as *, relative to the highest estimation under consideration) were determined using the Student’s t-test, p < 0.05

The RuBisCO activity was analyzed by adding the cofactor, NADH; the organic carbon substrate, RuBP; and the inorganic carbon (C_*i*_) substrate (NaHCO_3_, a fully soluble form; or CO_2_, relatively less soluble in liquid phase). The reaction rate (as µmol of RuBP consumed/min) and the RuBisCO activity (as nmol of NAD^+^/µg of Chl-a/min) were monitored with different crude protein loadings from the cell lysate (50–200 µg). By plotting absorbance versus NADH concentration, the standard curve for NADH was generated (Additional file 1: Fig. S2), for corresponding quantitation. We observed the highest rate of RuBP consumption of 0.0159 mmol/min for the reactions incubated with 5% CO_2_, whereas it was 0.0038 mmol/min for the bicarbonate supplemented reactions, with 200 µg of the protein. The reaction rates were observed to improve from 50 µg to 200 µg of protein loading. However, it was also observed that the increase in the protein amount did not increase the reaction rate proportionally (Fig. 2C). This is most likely due to the fact that the substrate concentration was not increased along with the enzyme concentration. It was evident that at all the protein loadings, reaction rates were 4–8 times higher with 5% CO_2_ than those supplemented with 20 mM NaHCO_3_ (Fig. 2A–C) [23–25]. Importantly, only 5–7% of CO_2_ is released from bicarbonate at physiological pH of 7–7.5 and more at lower pH [25]. Secondly, an additional step catalyzed by carbonic anhydrase is required for converting HCO_3_^−^ to CO_2_ [26]. Being out from the cellular microenvironment, i.e., in the cell lysate, could affect the caboxysomal interlinks between carbonic anhydrase and RuBisCO, consequently affecting the C-fixation from HCO_3_^−^[27]. Hence, both carbonic anhydrase activity and the reaction pH will concurrently affect the reaction rate with bicarbonate as a substrate [28]. Furthermore, the carbon availability in the liquid phase by 5% CO_2_ could be several folds higher than 20 mM NaHCO_3_. On the other hand, although NaHCO_3_ would have been more convenient inorganic carbon (C_*i*_) substrate for a biochemical assay, at higher concentrations it would influence the reaction pH, ultimately limiting the in vitro enzyme activity. In addition, at a low carbon dioxide-to-oxygen ratio RuBisCO enzyme binds to O_2_ which is a competitive substrate for CO_2_, [29]. Thus, utilizing 5% CO_2_ as a substrate instead of NaHCO_3_ increases the carbon dioxide-to-oxygen ratio which in turn reduces the chance of photorespiration to occur and improves the net rate of carboxylation in the assay.

We also calculated RuBisCO activity as nmol of NAD^+^/µg of Chl-a/min. Chlorophyll-a (Chl-a) concentrations were calculated for all the cell lysates. The highest RuBisCO activity (2.13 nmol of NAD^+^/µg of Chl-a/min) was observed with 50 µg of protein loading and was found to decrease with increase in protein loading. The activities of 1.66 nmol of NAD^+^/µg of Chl-a/min, 1.45 nmol of NAD^+^/µg of Chl-a/min, and 1.24 nmol of NAD^+^/µg of Chl-a/min were observed with protein loadings of 100 µg, 150 µg, and 200 µg respectively (Fig. 2D). The trend could primarily be attributed to the fact that the substrate concentration was not increased concomitantly with increase in protein loading [29]. In theory, by conducting detailed studies at higher protein loading it is possible to achieve the highest activity, currently achieved with lower protein concentration. Previously, based on the same principle, Rasmussen et al. had developed an NADH-dependent RuBisCO activity assay using permeabilized cyanobacterial cells, where for PCC 6803 they measured a RuBisCO activity of 5.2 nmol of NADH/ µg of Chl-a/min [16]. However, unlike this assay (Additional file 1: Table S1), their assay reaction was externally supplemented with the pathway enzymes, substrates, and cofactors. Overall, although the assay developed in this study was based on the procedure by Rasmussen et al. [16], we have simplified it by using the cell’s native enzymes, to ensure convenient implementation, reproducibility, reduced cost, and to avoid any manual interventions and errors due to the use of external enzymes from different sources and manufacturers. Secondly, their use of permeabilized cells might still restrict the mass transfer of reactants and products across the cell membrane. Hence, under current work, the cell lysate has been used as the source of different native enzymes and intermediate metabolites, contributing to the RuBisCO assay. Importantly, this simplified new assay provided the activity with the same order of magnitude as reported in the literature (Additional file 1: Table S2).

### Fluorescence based in vitro RuBisCO activity assay

The NADH-dependent biochemical assay developed in the previous section can be employed to study RuBisCO activity. However, the NADH oxidation during the GAPN activity occurs two-steps downstream of RuBisCO catalyzed step (Fig. 1). To obtain the estimations closer to the RuBisCO catalyzed reaction, we developed another assay to track the kinetics of ATP hydrolysis catalyzed by 3-PGK, using 3-PG as a substrate, which is a direct product of RuBisCO catalysis. The reaction phosphorylates 3-PG to 1,3-BPG with concurrent ATP hydrolysis to ADP (Fig. 1). ADP generated during ATP hydrolysis can be recognized by a sensor, termed as Spinach-based ADP-sensor [30, 31], at an optimal pH and can impart fluorescence upon binding to DFHBI (Fig. 1). However, in the absence of the ligand (ADP) molecule the Spinach sensor remains un-folded and non-fluorescent.

**Fig. 3:**
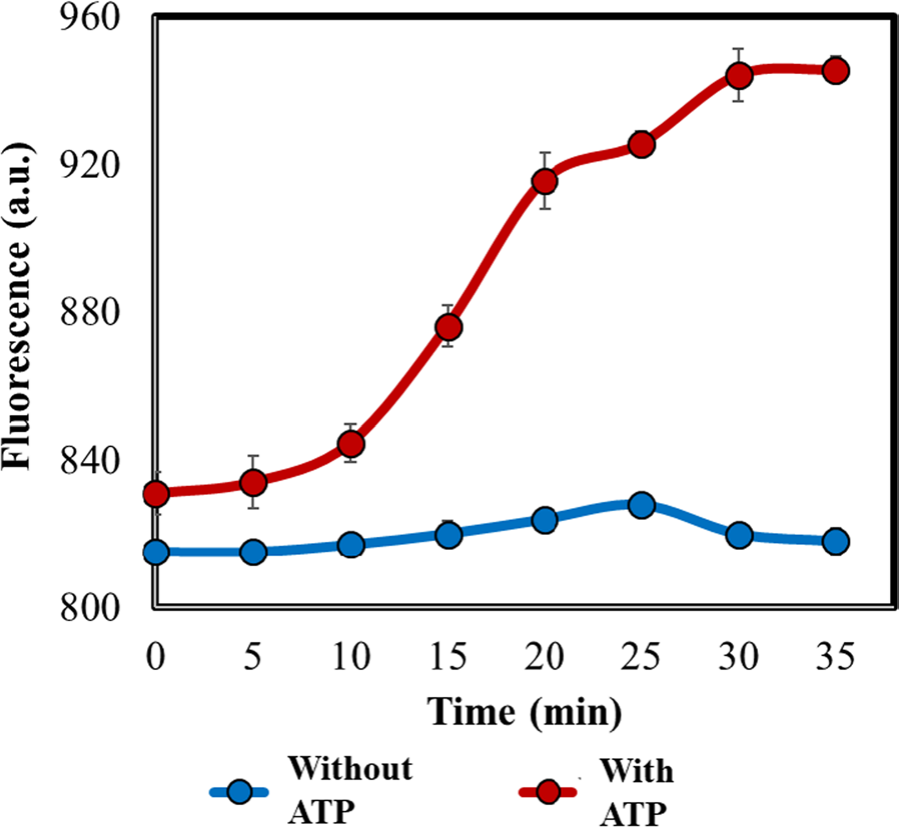
RNA aptamer-based in vitro RuBisCO activity assay. RNA aptamer-based RuBisCO activity assay for detecting ATP hydrolysis to ADP during 3-PG → 1,3-BPG was developed. The ADP-specific sensor was tested with the cyanobacterial cell lysate containing 100 µg of total protein with or without external ATP addition to the reaction mixture in HEPES buffer (pH 7.4). Comparative ATP hydrolysis exhibited significant increase in fluorescence in the reaction mixture containing ATP over the one without ATP supplementation. The assay was performed in biological triplicates

A polynucleotide (DNA) containing the sequences for P_*T7*_ and the RNA aptamer with an ADP recognizing module and a transducer module (Additional file 1: Sequence S1), was commercially synthesized. We PCR amplified it, and the purified polynucleotide was subjected to in vitro transcription (Additional file 1: Table S3). The generated RNA transcript was used as a sensor for sensing ATP hydrolysis catalyzed by 3-PGK. Preliminary screening was performed to verify the functioning of the RNA sensor, by directly supplementing ADP in the reaction mixture containing DFHBI in HEPES buffer (pH 7.4). The reaction curve displayed an increase in the fluorescence indicating positive response of the RNA sensor to the presence of ADP (Additional file 1: Fig. S3A). Simultaneously, three different reaction controls (with ATP and RuBP, without incubating under 5% CO_2_; with ATP, without RuBP; and with RuBP, without ATP: both incubating under 5% CO_2_) were analyzed. No signals in these control assays further confirmed the specificity of the RNA sensor for ADP, and negligible ADP contribution from other metabolic pathways to the reaction fluorescence (Additional file 1: Fig. S3B). Having verified that, a separate set of experiment was conducted under 5% CO_2_, with the assay mixture containing RuBP, DFHBI and the cell lysate corresponding to 100 µg of the protein in HEPES buffer (pH 7.4). A comparative analysis of the fluorescence signals from the ‘test’ reaction containing 2 mM ATP and the ‘control’ reaction without ATP was performed. The results distinctly indicated that the fluorescence signal steeply rose for the test sample, whereas there was no increase in the signal for the control (Fig. 3). Advantageously, as the RNA aptamer can be expressed in vivo, it can be used for in vivo fluorescence analysis as demonstrated in the next section, and can be effectively extended for high throughput screening [32, 33].

### Fluorescence based in vivo RuBisCO activity assay

**Fig. 4:**
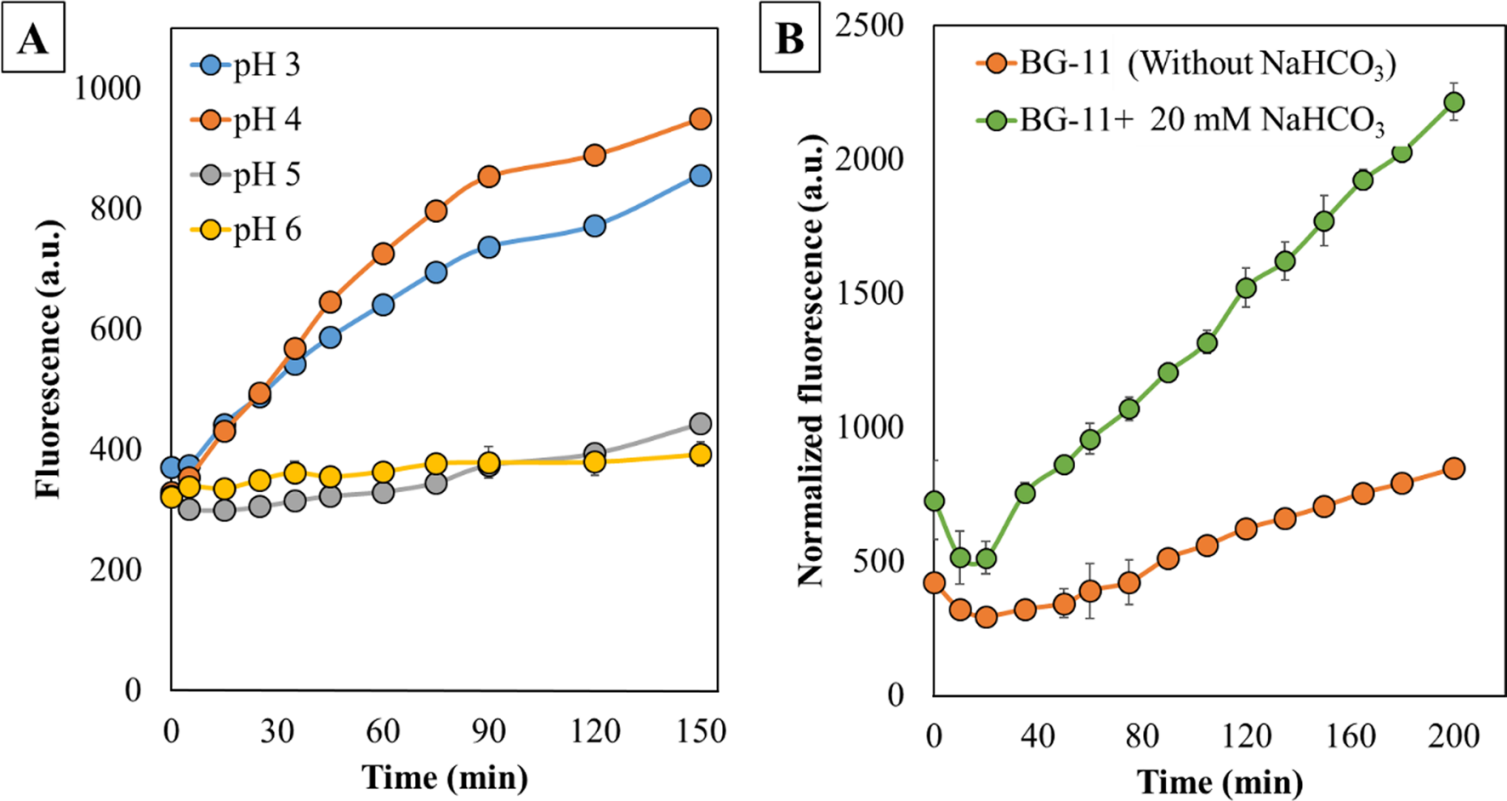
In vivo ADP-specific sensor based fluorescence assay. **A** The recombinant strains of PCC 6803 (SSL142) were grown with atmospheric CO_2_ and induced in BG-11 + 50 µg/mL of kanamycin were subjected to the fluorometric assay with different pH (3, 4, 5 or 6) citrate buffers. Their comparative analysis over 150 min indicated a significant increase in the fluorescence at pH 4 followed by 3, 5 and 6. **B** SSL142 was grown in BG-11 + 50 µg/mL of kanamycin, with and without 20 mM of NaHCO_3_. Both the cultures were subjected to the fluorescence assay at pH 4. Fluorescence values were normalized with the corresponding OD_730_, where the results indicated three-fold higher rate of increase in the fluorescence with SSL142 grown in presence of NaHCO_3_. All the assays were performed in biological triplicates

With an aim of demonstrating the feasibility of ADP-specific sensor’s in vivo application for monitoring RuBisCO activity, the wild-type (WT) strain of PCC 6803 was transformed with a replicative plasmid, pNH-RNA containing a nucleotide sequence for ADP-specific sensor, under an IPTG inducible P_*trc*_ promoter. As a first step, in vivo performance of the RNA sensor was analyzed in *Escherichia coli*. Towards this, the *E. coli* strain DH5α and its recombinant strain (SSL141) expressing the RNA aptamer were subjected to fluorescence assay in a reaction mixture with the HEPES buffer (pH 7.4). SSL141 exhibited a prominent increase in fluorescence owing to the non-photosynthetic ADP contributing reactions, while there was no change in fluorescence for *E. coli* DH5α (Additional file 1: Fig. S4A, S4B). However, when this assay was performed with WT and the recombinant strain of PCC 6803 (SSL142), SSL142 did not exhibit increase in fluorescence as was observed with SSL141, in the reaction buffer at pH 7.4. The assay was also repeated with the spent BG-11 medium, that had an alkaline pH 9–10, but still no significant increase in the fluorescence was observed (data not shown). As pH is a critical factor influencing the C_*i*_ availability and the aptamer folding, the assay for SSL142 strain was further performed in the 0.1 M citrate buffer maintained at a pH 3, 4, 5 or 6. Interestingly, fluorescence increase was observed at the pH of 3 and 4 (Fig. 4A). However, the fluorescence decreased as the pH was decreased from 4 to 3. It is likely that a very low pH of 3 was deleterious to the cell and/or enzymes [34]. A comparative analysis was carried out between WT and SSL142 at pH 4, to ensure and demonstrate the reproducibility of this novel assay. As expected, SSL142 exhibited increase in fluorescence, whereas there was no increase in fluorescence for the WT for the entire duration of the experiment (Additional file 1: Fig. S4C).

Finally, to prove that the fluorescence assay can be used to analyze the RuBisCO activity, SSL142 was grown in BG11 medium supplemented with and without 20 mM NaHCO_3_ and induced for expressing the RNA aptamer. Significantly higher rate of fluorescence was observed in the cells grown with NaHCO_3_ (Fig. 4B). As the presence of NaHCO_3_ increases the flux through the Calvin cycle, which in turn would increase ATP hydrolysis, this increase in fluorescence corresponded to the in vivo RuBisCO activity [35, 36]. These results suggested that the performance of an RNA aptamer for in vivo assays would be significantly influenced by the intracellular pH, cultivation conditions, type of organism as well as the type of metabolic engineering which could contribute to the fluorescence through overexpression of the enzymes involved with ATP hydrolysis. In addition, the aptamer sensitivity for the corresponding substrate or the cofactor would also play a crucial role while extending it for quantitative assays. However, contemplating the in vivo performance of the RNA aptamer, the approach can be easily extended for high-throughput screening for efficient clonal selection [32].

### Comparative statistical analysis of the RuBisCO activity assay

**Fig. 5:**
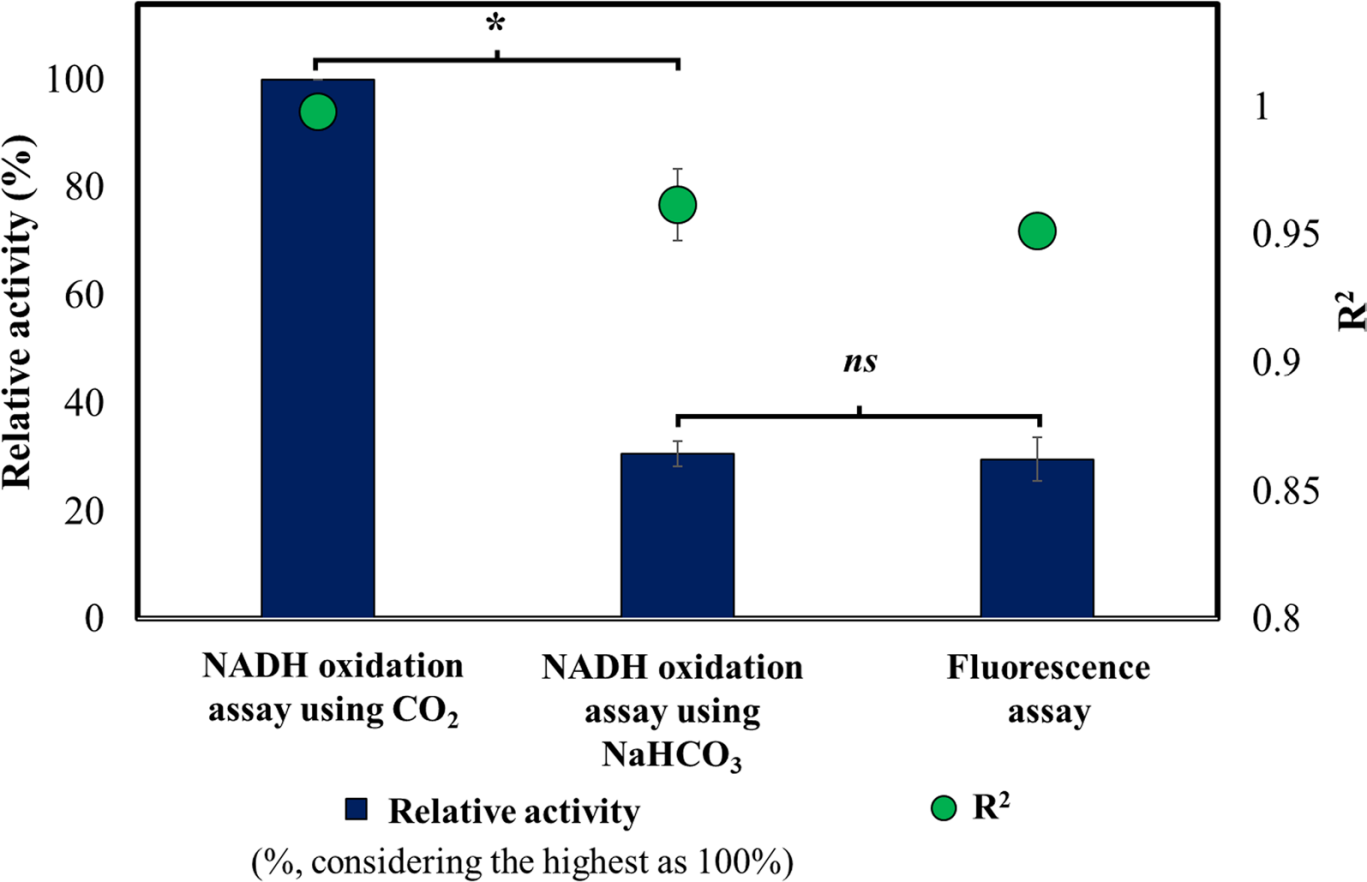
Comparative analysis of biochemical and fluorescence based assays. Results from the NADH-based biochemical assays using bicarbonate and CO_2_ as inorganic C substrates, and the RNA-aptamer based in vitro fluorescence assays, were normalized as percent relative activities. The highest activity obtained with the biochemical assay using 5% CO_2_ was normalized to be 100% (the bars). The corresponding correlation coefficients were compared for validating the methods, showing them in the range 0.95–1.00 (the dots), indicating strong correlation corresponding to increase in the activity with time. All the assays were performed in biological triplicates and statistically significant differences were obtained using the Student’s t-test, p < 0.05. *statistically significant difference; *ns*, no significant difference

All the three in vitro RuBisCO assays were compared based on their percentage relative activity (% relative activity) and the correlation coefficient (R^2^ value). The results exhibited that the biochemical assay using 5% CO_2_ as a substrate had the highest % relative activity (considered to be 100%) than the other two assays (30.70% for the assay using 20 mM NaHCO_3_ as a substrate, 29.64% for the in vitro fluorescence-based assay) (Fig. 5). Although the in vitro fluorescence-based assay targeted ADP, which was obtained from ATP hydrolysis during 1,3-BPG synthesis from 3-PG (the product of RuBisCO catalysis), the fluorescence signal was generated only upon ADP binding, followed by the DFHBI binding to the ADP-sensor. These would be additional non-biochemical steps that could contribute to a lag in this assay [30, 31]. Therefore, ADP binding to the aptamer and generation of fluorescence signal could itself be rate-limiting than ATP hydrolysis to ADP. In addition, pH values above 8.0 are found to be optimal for RuBisCO activation and catalysis [37, 38]; whereas the optimal pH for effective RNA aptamer folding and activation was 7.4 [30, 39]. These different pH optima could also be one of the contributing factors for its slower rate than the biochemical assays and faster signal saturation (Fig. 3). Hence, a separate study can be conducted to address this bottleneck, for example by checking the influence of variable DFHBI concentrations on the rate of reaction or using another aptamer exhibiting closer pH optima to that for RuBisCO activation.

In addition, to validate the accuracy of the methods, correlation coefficients (R^2^) were obtained from the linear range of the activity plots (n = 3). The results showed that R^2^ was in the range of 0.95–1.00, establishing a very strong correlation in the linear range, as the reaction progresses [40] (Fig. 5). Assays were repeated in biological triplicates and their reproducibility was analyzed using the Student’s t-test. The *p*-values showed no significant difference between them (Additional file 1: Table S4), indicating strong reproducibility of the assays. Based on their comparative statistical analyses, fluorometric assays would be more suitable for qualitative or semi-quantitative estimations whereas NADH-based spectrophotometric biochemical assay could be prominently exploited during quantitative analysis.

## Conclusion

In conclusion, under current investigation, we developed a convenient and easily reproducible NADH-dependent RuBisCO activity assay for its quantitative evaluations. The assay was predominantly reliant on cellular reaction components, and minimizing the need for external biochemical (enzymes or metabolite) additions. This rendered it cheaper and simpler compared to the existing assay strategies. Additionally, we developed a novel approach for in vivo monitoring of the RuBisCO activity, using RNA aptamer-based fluorescence sensor. By optimizing the external pH and NaHCO_3_ (C_*i*_) supplementation, we demonstrated that the RNA aptamer based ADP-specific sensor can be used for qualitatively monitoring the RuBisCO activity in vivo. This further indicated its potential for easy extension to high throughput screening and clonal selection. However, the in vivo assay reported here is confined to lower pH and therefore, further work is required to select or to improve the RNA sensor to monitor RuBisCO activity at pH above 7. Both the types of assays (biochemical and fluorometric) are platform-independent, rapid, and effective for translating to screen a wide range of RuBisCO variants in a time efficient manner. Moreover, since both these assays exploit NADH and ATP as the molecule to be detected, the strategies can be readily extended to investigate other biochemical reactions utilizing these biomolecules.

## Methods

### Reagents and chemicals

All chemicals including adenosine tri-phosphate (ATP), adenosine di-phosphate (ADP), β-nicotinamide adenine dinucleotide, reduced disodium salt (NADH), ribulose 1,5-bisphosphate (RuBP), (*Z*)-5-(3,5-Difluoro-4-hydroxybenzylidene)-2,3-dimethyl-3,5-dihydro-4*H*-imidazol-4-one (DFHBI), protease inhibitor cocktail (EDTA free), and RNaseZAP™ were purchased from Sigma Aldrich, MO, USA. The *in-vitro* transcription kit Ampliscribe™ T7-*Flash*™ was purchased from Epicentre (obtained through Lucigen^®^). All the components of the cell culture medium were purchased from Sigma-Aldrich, MO. Bradford’s reagent was procured from BioRad Inc., USA.

### Preparation of crude cell lysate

The wild-type strain of PCC 6803 was cultivated at 30 °C under 55 µmol m^−2^ s^−1^ of light in a liquid Blue Green-11 medium (BG-11). Cell growth was monitored by measuring the optical density at 730 nm (OD_730_) with an Infinite 200 PRO microplate reader (Tecan). 20 mL of exponentially growing cell culture (OD_730_ = 0.4) was centrifuged at 5000 rpm for 20 min, the supernatant was discarded, and the cell pellet was used for further studies. The cell pellet was frozen at -80 °C for 2 h for improving the cell lysis [41]. The cell pellet was suspended in 300 µL of 100 mM Tris–HCl buffer (pH 8.0) and 3 µL of protease inhibitor cocktail (EDTA free) was added into it. Equal volume of glass beads (0.1 mm, from Scientific Industries, Inc. International) were added to the cell suspension and the resulting mixture was vortexed for 25–30 min at room temperature to disrupt the cells. The vortexed mixture was centrifuged again for 5 min at 4000 x g. The supernatant (cell lysate) was collected, and its protein concentration was determined using Bradford’s assay. Briefly, 5 µL of the lysate and 250 µL of Bradford’s reagent were mixed and incubated in dark for 5 min. Absorbance was recorded at 595 nm and the protein concentration was quantified using a standard curve (Additional file 1: Fig. S5). The crude cell lysate was stored on ice for conducting enzymatic assays.

For calculating the RuBisCO activity, chlorophyll-a (Chl-a) concentrations in corresponding cell pellets were estimated. The pellet was suspended in 1 mL of chilled absolute methanol and incubated at 4 ℃ for 20 min. The mixture was centrifuged at 15,000 x *g* for 1 min and the absorbance of the supernatant was estimated at 665 nm and 720 nm, keeping methanol as blank. The Chl-a concentration was calculated as follows [42]:

$${\text{Chl - a }}\left( {\mu {\text{g}}/{\text{mL}}} \right){\mkern 1mu} = 12.9447 \times {\mkern 1mu} \,\left( {{\text{A}}_{{665}} - {\text{A}}_{{720}} } \right)$$

### Absorbance based assay

A reaction mixture containing 15 mM MgCl_2_, 1 mM EDTA, 2 mM ATP, 50 mM Tris–HCl (pH 8.0), and crude protein extract was incubated for 20 min with 20 mM NaHCO_3_ or in an incubator with 5% CO_2_ at 30 ℃, with continuous shaking at 200 rpm. A pre-incubation step is necessary to ensure carbamylation and activation of RuBisCO present in the cell lysate [1]. Following pre-incubation with 5% CO_2_ and 55 µmol m^−2^ s^−1^ light at 30 ℃, 0.5 mM NADH and 0.5 mM RuBP were immediately added into the reaction mixture (Additional file 1: Table S1). NADH oxidation rate was continuously monitored by measuring the absorbance at 340 nm using an Infinite M NANO^+^ (Tecan) plate reader maintained at 30 ℃ [16]. RuBisCO activity was defined as nmol of NAD^+^ formed (positive slope) or NADH oxidized (negative slope) per mole of chlorophyll-a (active photosynthetic reaction center) per minute and was calculated in the linear range of the plot (Abs_340_ vs time, in min). Steps to calculate the RuBisCO activity (nmol of NAD^+^ produced/ µg of Chl-a/ min) have been given in Additional file 1: Protocol S1. RuBP consumption rate was calculated using the following formula;

$${\text{RuBP}}\,{\text{consumption}}\,{\text{rate}}\,\left( {\mu {\text{mol}}/{\text{min}}} \right) = \frac{{{\text{Slope}}\left( {\frac{{{\text{Abs}}340}}{{{\text{min}}}}} \right) \times\,{\text{Reaction}}\,{\text{volume}}\,\,\left( {{\text{mL}}} \right)}}{{{\text{Molar}}\,{\text{extinction}}\,{\text{coefficient}},\,\varepsilon \left( {\frac{{{\text{mL}}}}{{\mu ({\text{mol}}}.{\text{cm})}}} \right) \times \,{\text{NADH}}\,{\text{factor}}\,{\text{X}}\,{\text{Pathlength}}\,\,\left( {{\text{cm}}} \right)}}$$

where, the reaction volume was 0.2 mL, ε was 6.22, NADH factor (no. of NADH molecules oxidized per molecule of RuBP) was 2, and the pathlength was 0.574 cm [5]. Furthermore, the relative (%) activities were calculated considering the highest RuBisCO activity to be 100%. Appropriate control experiments were conducted to ensure NADH as a compatible cofactor for the reaction and significant contribution from photosynthetic NADH oxidation than other metabolic reactions (Additional file 1: Fig. S1).

### RNA sensor synthesis

The polynucleotide sequence for the Spinach-based ADP-specific sensor along with upstream P_*T7*_ promoter sequence (Additional file 1: Sequence 1A) was synthesized by Integrated DNA Technologies, USA [30, 31]. This polynucleotide fragment was PCR amplified using P_*T7*_ forward primer (taatacgactcactataggg) and ADP-R reverse primer (ctccgtaactagtcgcgtc). The amplified fragment was gel purified, the DNA concentration was determined using Nanodrop ND-1000 (Thermo Scientific) and used as a template for in vitro transcription. In vitro transcription was performed by using commercially available Ampliscribe™ T7-*Flash*™ transcription kit (Epicentre) to obtain the Spinach-based ADP-specific sensor. The protocol for in vitro transcription and purification of the Spinach-based sensor is mentioned in Additional file 1: Table S3). The linear DNA template, 100 mM of each of the NTPs, RNAse inhibitor and T7 RNA polymerase were mixed as directed to the final volume of 20 µL, followed by incubation at 30 ℃ for 30 min. The transcript thus generated was purified using ammonium acetate precipitation method [43]. The in vitro transcription mixture was mixed with one volume (20 µL) of 5 M ammonium acetate. Incubated on ice for 15 min and centrifuged at 10,000 x g for 10–15 min. Pellets of RNA were washed with 70% ethanol and stored at − 80 ℃ [43]. All the plasticwares and pipettes were treated with RNaseZAP™ for all RNA related experiments to render them nuclease (RNase) free.

### Activation of the RNA based ADP-specific sensor

The purified Spinach-based ADP sensor (1 µM) was incubated with 40 mM HEPES buffer (pH 7.4), 1 mM EDTA, 10 µM DFHBI, 15 mM MgCl_2_, and 125 mM KCl with continuous shaking (200 rpm) at 37 ℃ for 10 min for proper folding of Spinach-based ADP sensor. 1 mM ADP was added into the reaction mixture; the sensor has a recognition site for the ligand ADP molecule. When a ligand molecule binds with the sensor, it induces the folding of Spinach portion of the sensor, thereafter DFHBI binds with it and activates fluorescence [31]. The fluorescence intensity was recorded immediately after adding 1 mM ADP solution for a time period of 50 min with 5 min interval at an excitation wavelength of 460 nm and emission at 510 nm.

### In vitro RuBisCO activity assay using Spinach-based ADP-specific sensor

100 µL of a reaction mixture (containing 15 mM MgCl_2_, 1 mM EDTA, 2 mM ATP, 40 mM HEPES buffer (pH 7.4), 10 µM DFHBI, 1 µM RNA sensor, 125 mM KCl, and 0.5 mM RuBP were mixed with 100 µg of the crude protein extract. The reaction mixture containing crude protein extract was transferred to a CO_2_ incubator which was maintained at 5% CO_2_ throughout the duration of the experiment. The reactions were performed in a 96-well black-walled microplate (Corning) with continuous shaking at 200 rpm and fluorescence was monitored at an excitation wavelength of 460 nm & emission at 510 nm [31]. Appropriate control experiments were conducted to ensure that significant contribution for fluorescence is resulting from photosynthetic ATP hydrolysis as compared to other metabolic reactions (Additional file 1: Fig. S3).

### Development of recombinant strains for in vivo ADP-specific sensor based fluorescence assay

pNH_RNA plasmid got synthesized from Twist Biosciences, by cloning the gene for RNA aptamer in pBb(RSF1010)-1 k GFPuv plasmid (Addgene No. 106395), under the control of P_*trc*_ promoter, by replacing the GFP gene, in *E. coli* DH5α. The sequence has been provided in the Additional file 1: Sequence S1B). The plasmid was transformed in the wild-type (WT) strain of PCC 6803, using the protocol by Zang et al. [44]. Positive colonies were identified through colony PCR. The recombinant strains were grown by adding 50 µg/mL kanamycin to the corresponding culture media. *E. coli* DH5α and the recombinant strain, SSL141 were grown in hybrid M9 medium at 37 ℃ [45]. 1% of the overnight grown cultures were freshly inoculated in 5 mL hybrid M9 medium at 37 ℃. The culture growth was monitored and the cultures were induced with 1 mM IPTG at the OD_600_ of 0.6 and further incubated overnight. Similarly, the recombinant (SSL142) and WT strains of PCC 6803 were grown in BG-11 medium at 30 ℃, under the light intensity of 80 µmol m^−2^ s^−1^. 1% of the actively growing cultures were freshly inoculated in 20 mL BG-11 media at 30 ℃, under the light intensity of 80 µmol m^−2^ s^−1^. The culture growth was monitored and the cultures were induced with 1 mM IPTG at the OD_730_ of 0.6 and further incubated overnight. All the four cultures were subjected to the in vivo fluorescence assay.

### In vivo RuBisCO activity assay using Spinach-based ADP-specific sensor

All the four cultures (*E. coli* DH5α, SSL141, WT PCC 6803 and SSL142) with the final OD of 1.0 were suspended in 100 µL of reaction buffer containing 15 mM MgCl_2_, 1 mM EDTA, 125 mM KCl and 10 µM DFHBI in 40 mM HEPES buffer (pH 7.4). As no fluorescence change was obtained for the SSL142, four different reaction buffers, using pH 3, 4, 5 and 6 citrate buffer (0.1 M), were prepared and analyzed. For in vivo assay, the SSL142 strains were grown in BG-11 medium with or without the supplementation of 20 mM NaHCO_3_, and 50 µg/mL kanamycin. Cells were harvested during the mid-exponential phase and subjected to the assay conditions. The results were compared based on the fluorescence normalized with the corresponding culture OD_730_. All the reactions were performed in a 96-well black-walled microplate (Corning) under shaking at 200 rpm and intermittent (after every spectrophotometric reading) exposure to 80 µmol m^−2^ s^−1^ of light at 30 ℃, in biological triplicates. The fluorescence was monitored at an excitation wavelength of 460 nm and emission at 510 nm [31].

### Supplementary Information

**Additional file 1: Figure S1.** In vitro biochemical assay: Preliminary experiments were performed to estimate the effectiveness of using the cell’s native enzymes for performing the NADH-based spectrophotometric RuBisCO activity assay. The assay was performed by externally supplementing the reaction with two crucial substrates; RuBP and NADH. Simultaneously, two reaction controls were introduced; **a** without RuBP, with NADH, and **b** without NADH, with RuBP. The results showed in the plot indicated that there was negligible or no increase in the mM of NAD^+^ (product of NADH oxidation) for the two controls, where the test sample containing both the substrates showed prominent increase in NAD^+^ concentration, confirming the in vitro functionality of the assay. The graph for the control reaction without RuBP, further indicated that contribution from the other metabolic pathways to the NADH oxidation was relatively low and below the detection limit of the assay. **Figure S2.** Standard curve for NADH concentration vs Absorbance at 340 nm. **Figure S3.** Analysis of the functioning of the ADP-specific RNA sensor. **A** Functioning of the ADP-specific RNA sensor was confirmed by directly adding 2 mM ADP to the reaction mixture containing the HEPES buffer (pH 7.4) and in vitro transcribed RNA sensor, followed by monitoring its fluorescence. Increase in the fluorescence (subtracting 0 min fluorescence) confirmed the positive response of the sensor to ADP. **B** Background fluorescence or ADP contribution from other metabolic reactions was analyzed using the control assays. The assay mixture contained 0.5 mM RuBP as the first RuBisCO substrate, 2 mM ATP as the cofactor, and cell lysate as the source of pathway enzymes, where the plate was incubated in a CO_2_ incubator with 5% CO_2_ (second inorganic C substrate for RuBisCO). To prove the effectiveness of the assay, three different controls were introduced a) with ATP and CO_2_, but without RuBP, b) with RuBP and CO_2_, but without ATP, and c) with ATP and RuBP, but without incubating in 5% CO_2_. The results showed in the plot indicated that there was no increase in the fluorescence for the three controls. No increase in the fluorescence for the control reactions without RuBP, and without 5% CO_2_ incubation, further indicated that ADP contribution from the other metabolic pathways to the fluorescence due to ATP hydrolysis was below the detection limit of the assay. Similar results in the third control assay without ATP confirmed the specificity of the RNA aptamer for ADP. **Figure S4. **In vivo ADP sensor assay. **A** Preliminary verification for estimating the expression of ADP sensor and monitoring its fluorescence in non-recombinant (DH5α) and recombinant (SSL141) *E. coli* strains, where the results indicated positive increase in the fluorescence with SSL141 at pH 7.4. **B** Reproducibility of the assay was confirmed in the recombinant *E. coli*, in biological triplicates. **C** PCC 6803 WT and recombinant (SSL142) strains were grown under atmospheric CO_2_ conditions. ADP sensor expression and working was monitored in both the strains at the optimized reaction pH 4, where SSL142 exhibited increase in the fluorescence over WT. **Figure S5.** Standard curve for protein contents estimation. Standard curve was obtained by performing Bradford’s protein assay. Bovine serum albumin (BSA) was used as standard. Protein concentration of unknown sample was calculated from linear regression equation y = 0.1286x – 0.0095 obtained from the above plot. **Protocol S1. Table S1.** Assay reaction mixture. **Table S2.** Comparison of current RuBisCO activity with previously reported activities. **Table S3. **Protocol for in vitro transcription and purification of RNA. **Table S4.** Statistical analysis for determining reproducibility of the assay. All the assays were repeated in biological triplicates to determine their reproducibility. The results indicated that for all the assays, *p* > 0.05, indicating no significant difference between the activity values obtained while reproducing the assays. This confirmed the assay reproducibility. **Sequence S1. **Spinach based ADP sensor nucleotide sequence for in vitro studies. **Sequence S2. **Spinach based ADP sensor nucleotide sequence for in vivo studies.

## Data Availability

All data generated and analyzed during this study are included in the main manuscript and its Additional file 1.
