# Supplementary material for: RuBisCO activity assays: a simplified biochemical redox approach for in vitro quantification and an RNA sensor approach for in vivo monitoring
Source: Microb Cell Fact. 2024 Mar 14;23:83. doi: 10.1186/s12934-024-02357-6 (PMC10938803; doi:10.1186/s12934-024-02357-6)
Supplement: Supplementary file 1 — Additional file 1: Figure S1. In vitro biochemical assay: Preliminary experiments were performed to estimate the effectiveness of using the cell’s native enzymes for performing the NADH-based spectrophotometric RuBisCO activity assay. The assay was performed by externally supplementing the reaction with two crucial substrates; RuBP and NADH. Simultaneously, two reaction controls were introduced; a without RuBP, with NADH, and b without NADH, with RuBP. The results showed in the plot indicated that there was negligible or no increase in the mM of NAD+ (product of NADH oxidation) for the two controls, where the test sample containing both the substrates showed prominent increase in NAD+ concentration, confirming the in vitro functionality of the assay. The graph for the control reaction without RuBP, further indicated that contribution from the other metabolic pathways to the NADH oxidation was relatively low and below the detection limit of the assay. Figure S2. Standard curve for NADH concentration vs Absorbance at 340 nm. Figure S3. Analysis of the functioning of the ADP-specific RNA sensor. A Functioning of the ADP-specific RNA sensor was confirmed by directly adding 2 mM ADP to the reaction mixture containing the HEPES buffer (pH 7.4) and in vitro transcribed RNA sensor, followed by monitoring its fluorescence. Increase in the fluorescence (subtracting 0 min fluorescence) confirmed the positive response of the sensor to ADP. B Background fluorescence or ADP contribution from other metabolic reactions was analyzed using the control assays. The assay mixture contained 0.5 mM RuBP as the first RuBisCO substrate, 2 mM ATP as the cofactor, and cell lysate as the source of pathway enzymes, where the plate was incubated in a CO2 incubator with 5% CO2 (second inorganic C substrate for RuBisCO). To prove the effectiveness of the assay, three different controls were introduced a) with ATP and CO2, but without RuBP, b) with RuBP and CO2, but without ATP, and c) with ATP and [file 12934_2024_2357_MOESM1_ESM.docx]

**RuBisCO activity assays: a simplified biochemical redox approach for *in vitro* quantification and an RNA sensor approach for *in vivo* monitoring**

Muhammad Faisal^1,2,#^, Aditya P. Sarnaik^1,#^, Nandini Kannoju^1^, Nima Hajinajaf^1^, Muhammad Javaid Asad^2^, Ryan W. Davis^3^, Arul M. Varman^1^*

1. Chemical Engineering, School for Engineering of Matter, Transport and Energy (SEMTE), Arizona State University, Tempe, AZ, USA
2. University Institute of Biochemistry and Biotechnology, PMAS-Arid Agriculture University Rawalpindi, Pakistan 46000
3. Sandia National Laboratories, Livermore, CA, USA.

**
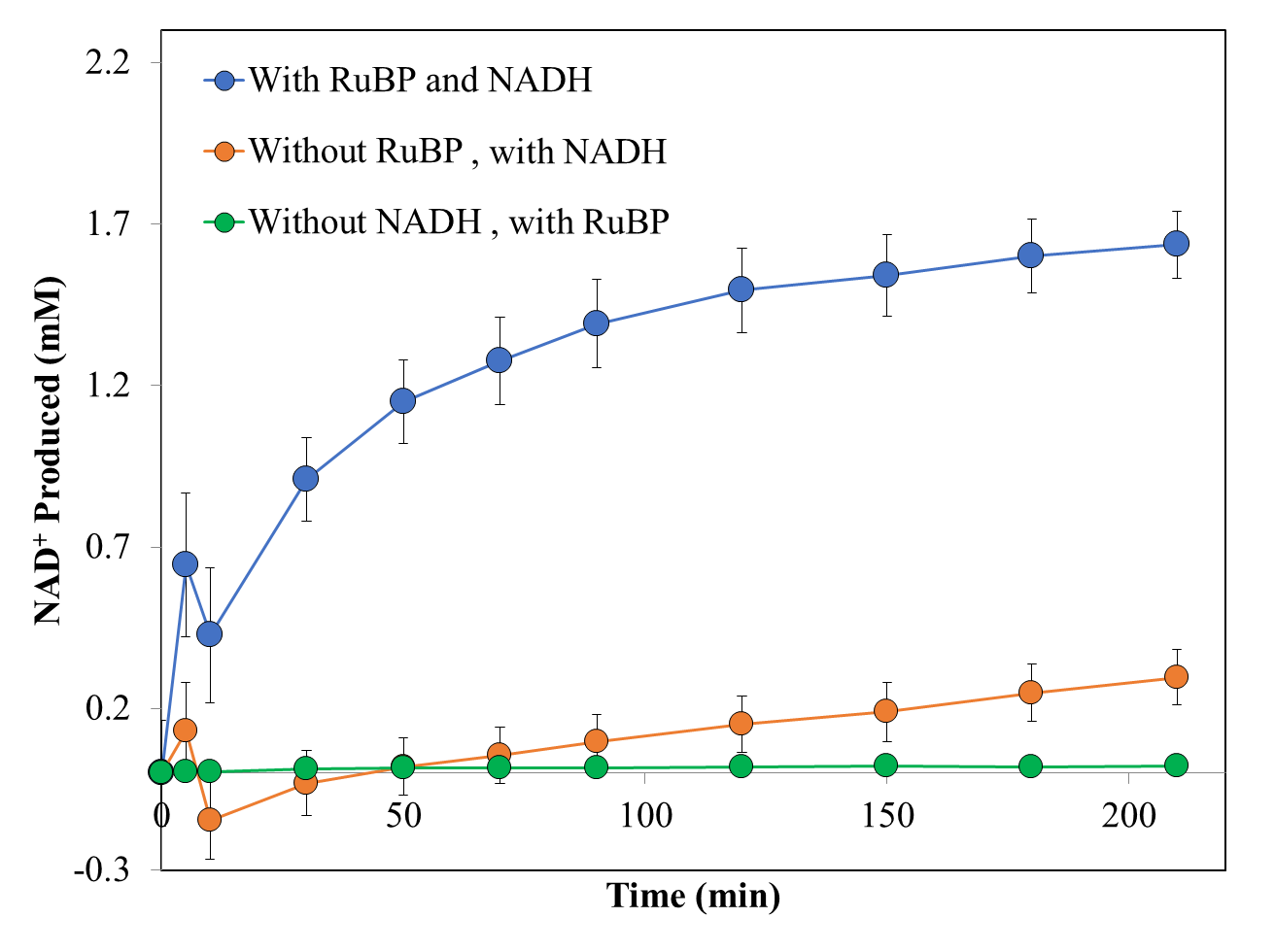
**

**Figure S1. *In vitro* biochemical assay**: Preliminary experiments were performed to estimate the effectiveness of using the cell’s native enzymes for performing the NADH-based spectrophotometric RuBisCO activity assay. The assay was performed by externally supplementing the reaction with two crucial substrates; RuBP and NADH. Simultaneously, two reaction controls were introduced; a) without RuBP, with NADH, and b) without NADH, with RuBP. The results showed in the plot indicated that there was negligible or no increase in the mM of NAD^+^ (product of NADH oxidation) for the two controls, where the test sample containing both the substrates showed prominent increase in NAD^+^ concentration, confirming the *in vitro* functionality of the assay. The graph for the control reaction without RuBP, further indicated that contribution from the other metabolic pathways to the NADH oxidation was relatively low and below the detection limit of the assay.

**Figure S2. Standard curve for NADH concentration vs Absorbance at 340 nm**

**
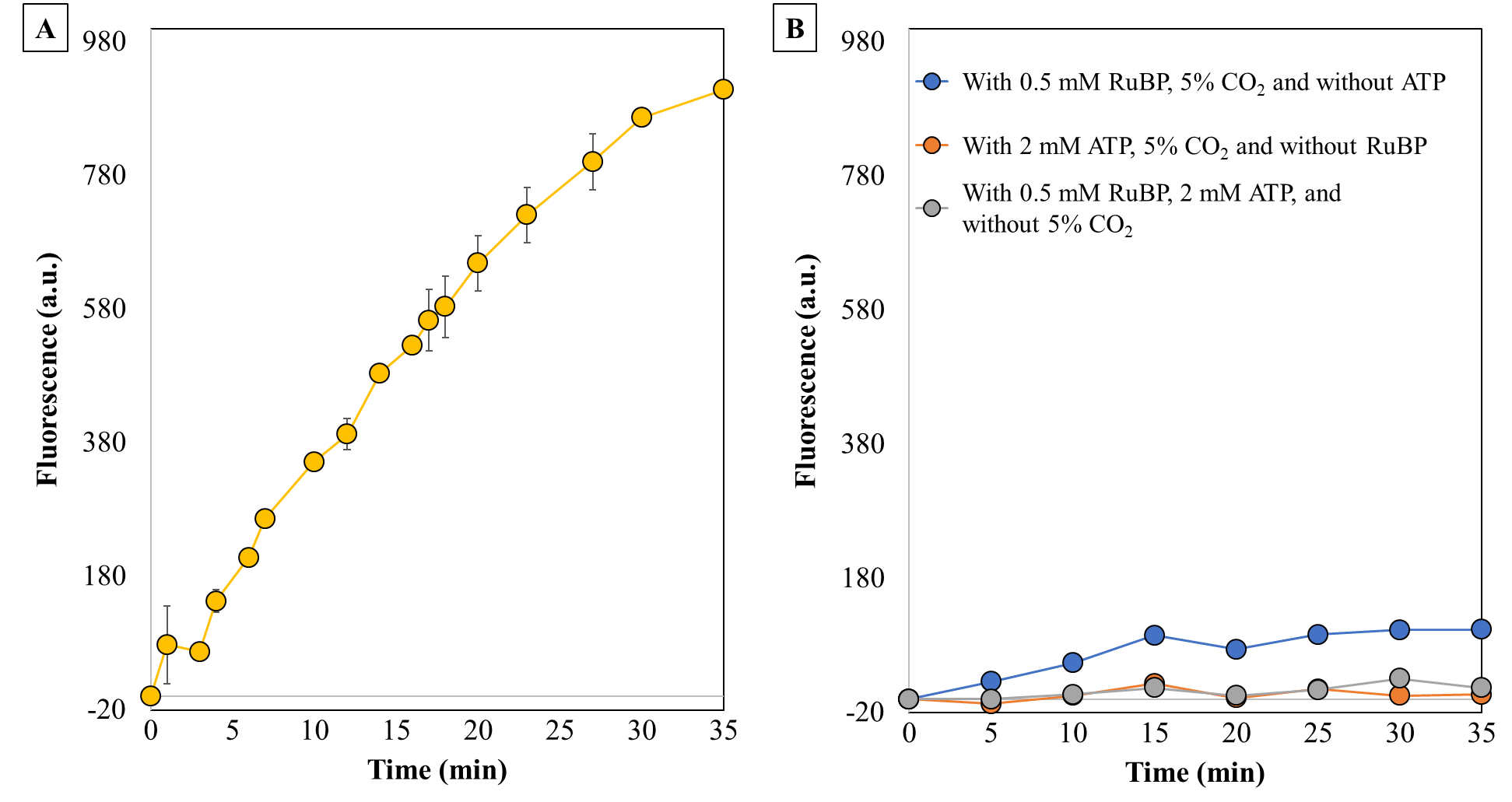
**

**Figure S3. Analysis of the functioning of the ADP-specific RNA sensor:** A) Functioning of the ADP-specific RNA sensor was confirmed by directly adding 2 mM ADP to the reaction mixture containing the HEPES buffer (pH 7.4) and *in vitro* transcribed RNA sensor, followed by monitoring its fluorescence. Increase in the fluorescence (subtracting 0 min fluorescence) confirmed the positive response of the sensor to ADP. B) Background fluorescence or ADP contribution from other metabolic reactions was analyzed using the control assays. The assay mixture contained 0.5 mM RuBP as the first RuBisCO substrate, 2 mM ATP as the cofactor, and cell lysate as the source of pathway enzymes, where the plate was incubated in a CO_2_ incubator with 5% CO_2_ (second inorganic C substrate for RuBisCO). To prove the effectiveness of the assay, three different controls were introduced a) with ATP and CO_2_, but without RuBP, b) with RuBP and CO_2_, but without ATP, and c) with ATP and RuBP, but without incubating in 5% CO_2_. The results showed in the plot indicated that there was no increase in the fluorescence for the three controls. No increase in the fluorescence for the control reactions without RuBP, and without 5% CO_2_ incubation, further indicated that ADP contribution from the other metabolic pathways to the fluorescence due to ATP hydrolysis was below the detection limit of the assay. Similar results in the third control assay without ATP confirmed the specificity of the RNA aptamer for ADP.


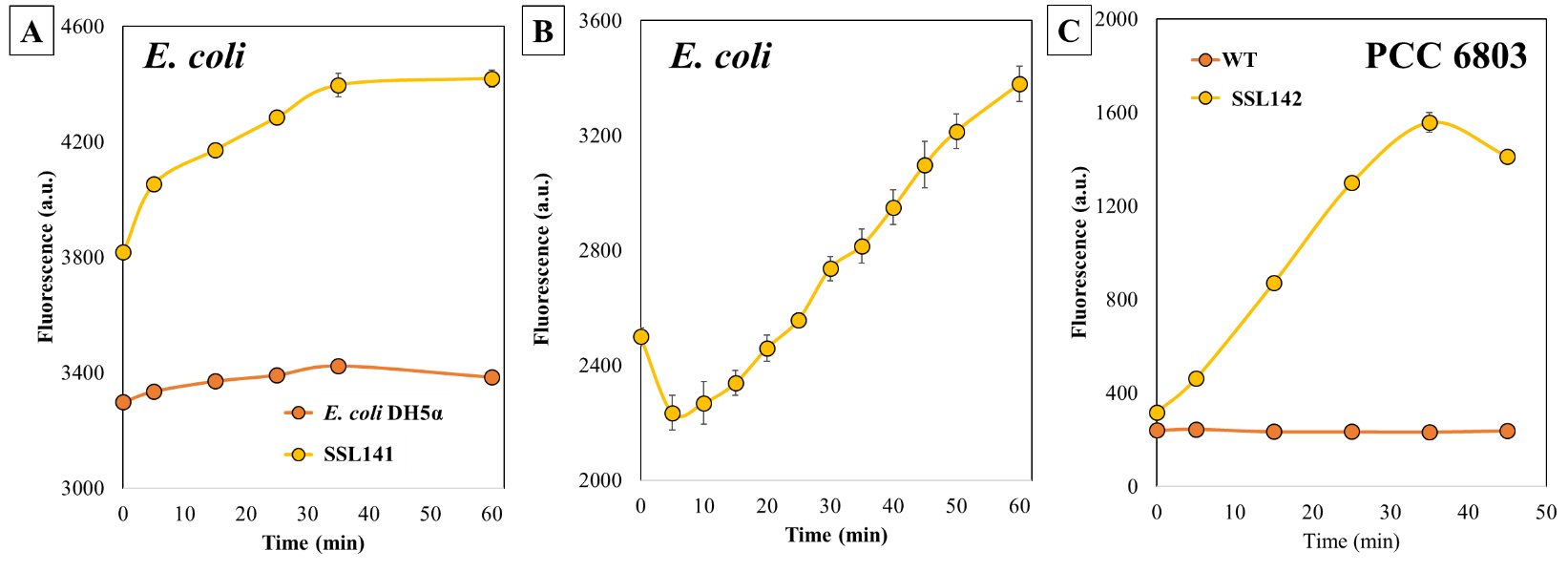


**Figure S4. *In vivo* ADP sensor assay:** A) Preliminary verification for estimating the expression of ADP sensor and monitoring its fluorescence in non-recombinant (DH5α) and recombinant (SSL141) *E. coli* strains, where the results indicated positive increase in the fluorescence with SSL141 at pH 7.4. B) Reproducibility of the assay was confirmed in the recombinant *E. coli*, in biological triplicates. C) PCC 6803 WT and recombinant (SSL142) strains were grown under atmospheric CO_2_ conditions. ADP sensor expression and working was monitored in both the strains at the optimized reaction pH 4, where SSL142 exhibited increase in the fluorescence over WT.

**Figure S5. Standard curve for protein contents estimation:** Standard curve was obtained by performing Bradford’s protein assay. Bovine serum albumin (BSA) was used as standard. Protein concentration of unknown sample was calculated from linear regression equation y = 0.1286x – 0.0095 obtained from the above plot.

**Protocol S1:**

**Step 1:** Take Abs_340_ at different time points for test (T_n_, with the cell lysate) and blank (B_n_, without the cell lysate) samples, corresponding to NADH oxidation. The experiment was performed for four different crude protein loadings (50, 100, 150 and 200 µg) from the lysate, in biological triplicates.

**Step 2:** Subtract T_n_ from B_n_ to estimate the actual NADH oxidation (in terms of A_340*_) due to RuBisCO activity.

**Step 3:** Estimate the corresponding NAD^+^ production as (A_340*(0 min)_-A_340*(n min)_) at all the time points, followed by converting it to the nmol using the standard equation.

**Step 4:** Simultaneously estimate the µg of Chl-a corresponding to µg of crude protein loadings from the lysate.

**Step 5:** Divide nmol of NAD^+^ estimated in step 3 by corresponding µg of Chl-a (nmol of NAD^+^ produced/ µg of Chl-a).

**Step 6:** Draw an XY-scatter plot for each set of readings obtained in Step 5 against time (min). Slope of the line in the linear phase of the graph will correspond to the RuBisCO activity nmol of NAD^+^ produced/ µg of Chl-a/ min.

**Step 7:** Calculate average slopes and corresponding standard deviations for making the comparative bar plot.

**Table S1. Assay reaction mixture**

| Chemicals | Required Conc. | Reaction Volume (μL) |
| --- | --- | --- |
| MgCl_2_ | 15 mM | 2 |
| EDTA | 1 mM | 2 |
| NADH | 0.5 mM | 2 |
| RuBP | 0.5 mM | 2 |
| Tris-HCl (pH-8.0) | 50 mM | x |
| NaHCO_3_ or  CO_2_ | 20 mM or  5 % | 2 or  x |
| ATP | 2 mM | 2 |
| Cell Extract | 100 μg | x |
| **Total Volume** |  | **200 μL** |

**Table S2. Comparison of current RuBisCO activity with previously reported activities**

| **References** | **RuBisCO Activity** | **Source of pathway enzymes** | **Methods** | **Strain** | |
| --- | --- | --- | --- | --- | --- |
| Rasmussen et al, 2016 | 4.0 ± 0.11 nmol NAD^+^.min^−1^.μg^−1^ Chl a | External source | NADH based assay with NaHCO_3_ | *Synechococcus* 7002 | 1 |
| Rasmussen et al, 2016 | 5.2 ± 0.17 nmol NAD^+^.min^−1^.μg^−1^ Chl a | External source | NADH based assay with NaHCO_3_ | *Synechocystis* 6803 | 2 |
| Stefanie & Miraj, 2014 | 455±30 nmol 3-PGA.min^−1^.mg^−1^ | External source | Radiometric ^14^C-based assay | *Thiomicrospira crunogena* | 3 |
| This Study | 2.13 nmol NAD^+^ .min^−1^.μg^−1^ Chl a. | Native enzymes | NADH based assay with 5% CO_2_ | *Synechocystis* 6803 | 4 |
| This Study | 0.8 nmol NAD^+^ min^−1^ μg^−1^ Chl a | Native enzymes | NADH based assay with 20 mM NaHCO_3_ | *Synechocystis* 6803 | 5 |

**Table S3. Protocol for *in vitro* transcription and purification of RNA**

1. Transcription reaction mixture

| Chemicals | Required Conc. | Reaction Volume  (μL) |
| --- | --- | --- |
| Linear template DNA with P_T7_ | 1 µg | x |
| Buffer | 10 X | 2 |
| ATP | 100 mM | 1.8 |
| CTP | 100 mM | 1.8 |
| GTP | 100 mM | 1.8 |
| UTP | 100 mM | 1.8 |
| DTT | 100 mM | 1.8 |
| RNase inhibitor |  | 0.5 |
| T7 RNA polymerase |  | 2 |
| Nuclease free water |  | x |
| **Total volume** | | 1. **μL** |

1. The volume of transcription reaction mixture was raised up to 20 μL with nuclease free water and incubated at 30 ^o^C for 30 minutes.
2. The ADP sensor was purified from transcription reaction mixture by ammonium acetate precipitation method. One volume of 5 M ammonium acetate (20 µL) was added to the *in vitro* transcription mixture (20 µL). Reaction mixture was incubated on ice for 15 minutes and centrifuged at 10,000 g for 10-15 minutes at room temperature. Then the purified RNA pellets were washed with 70% ethanol. ADP Sensor RNA pellets were stored at -80 ^˚^C. All the plastic wares and pipettes were treated with RNaseZAP^TM^ before any RNA purification experiments to render them nuclease (RNase) free.

**Table S4. Statistical analysis for determining reproducibility of the assay:** All the assays were repeated in biological triplicates to determine their reproducibility. The results indicated that for all the assays, *p* > 0.05, indicating no significant difference between the activity values obtained while reproducing the assays. This confirmed the assay reproducibility.

| **Assay** | | **Substrate** | **F value** | **F_crit_ values** | **t-test assuming__** | ***p* value (two-tailed test)** | **Confidence interval**  95%  ***p* value**  0.05 |
| --- | --- | --- | --- | --- | --- | --- | --- |
| *In vitro* assay | Biochemical | CO_2_ | 0.94 | 0.34 | Unequal variances | 2.085 |  |
|  |  | Bicarbonate | 0.97 | 0.37 | Unequal variances | 2.064 |  |
|  | Fluorometric | ADP | 1.18 | 4.88 | Equal variances | 2.179 |  |
| *In vivo* assay | Fluorometric | ADP | 1.02 | 3.18 | Equal variances | 2.101 |  |

**Sequence S1. Spinach based ADP sensor nucleotide sequence for *in vitro* studies**

TAATACGACTCACTATAGGGACGCGACTGAATGAAATGGTGAAGGACGGGTCCAGCACGAGGGGGAAACCCCGGACAATCAGACACGGTGCTTGTTGAGTAGAGTGTGAGCTCCGTAACTAGTCGCGTC

Red-T_7_ promoter sequence

Green- ADP recognition site

Black- Spinach RNA sequence with the green ADP recognition site

**Sequence S2. Spinach based ADP sensor nucleotide sequence for *in vivo* studies:** Considering the instability of Spinach-based sensor during in vivo monitoring, the sequence was incorporated in the tRNA scaffold (tRNALys), ensuring its stable expression, folding and fluorescence.

GGGGCCCGGATAGCTCAGTCGGTAGAGCAGcggGACGCAACTGAATGAAATGGTGAAGGACGGGTCCAGcacGAGGGGGAAACCCCGGACAATCAGACACGGtGcTTGTTGAGTAGAGTGTGAGCTCCGTAACTAGTCGCGTCccgCGGGTCCAGGGTTCAAGTCCCTGTTCGGGCGCCAtCTAGAGCGGACTTCGGTCCGCTTTTT

Yellow highlight: 5’ tRNALys sequence

Green highlight: 3’ tRNALys sequence

Blue highlight: terminator sequence

Green- ADP recognition site
